# Technical limitations and error propagation in the 3D printing pipeline for congenital heart disease: an integrative review—a narrative synthesis

**DOI:** 10.1093/ehjimp/qyag170

**Published:** 2026-11-05

**Authors:** Ayush Balaji, Sinling Tiffany Yu, Nabil Hussein, Tristan Ramcharan, Benjamin Pinkey, Antonio Fratini, Adrian Crucean

**Affiliations:** Birmingham Women’s and Children’s NHS Foundation Trust, Birmingham B4 6NH, UK; Hull York Medical School, University of Hull, Hull, UK; Birmingham Women’s and Children’s NHS Foundation Trust, Birmingham B4 6NH, UK; Aston University, Birmingham, UK; Birmingham Women’s and Children’s NHS Foundation Trust, Birmingham B4 6NH, UK; Birmingham Women’s and Children’s NHS Foundation Trust, Birmingham B4 6NH, UK; Birmingham Women’s and Children’s NHS Foundation Trust, Birmingham B4 6NH, UK; Birmingham Women’s and Children’s NHS Foundation Trust, Birmingham B4 6NH, UK; Aston University, Birmingham, UK; Birmingham Women’s and Children’s NHS Foundation Trust, Birmingham B4 6NH, UK

**Keywords:** 3D printing, congenital heart disease, quality assurance, medical image segmentation, geometric accuracy, surgical planning

## Abstract

Three-dimensional (3D) printing has gained prominence in the management of congenital heart disease (CHD) by augmenting spatial understanding, facilitating preoperative planning, and supporting education for clinicians and families. The clinical validity of printed models, however, is contingent upon accuracy maintained across a multistage pipeline that spans image acquisition, segmentation and modelling, printer-specific processing, and post-processing with subsequent clinical interpretation. Technical and human factors introduce discrepancies at each stage; these inaccuracies may accumulate and propagate, thereby altering the geometric and qualitative fidelity of the final model. This integrative review examines the principal sources of error across the pipeline, outlines mechanisms of propagation, and considers implications for decision-making in CHD, with particular emphasis on paediatric applications where permissible tolerances are narrow. Drawing upon contemporary literature, we provide recommendations for protocol standardization, validation procedures, operator training, and transparent documentation. An inter-observer experiment is proposed to quantify segmentation-induced variability using similarity and surface distance metrics, linking measurable divergence to potential clinical impact. A pragmatic error-minimizing pipeline is also described to improve reliability and promote reproducibility in clinical practice.

## Graphical Abstract

**Graphical Abstract:**
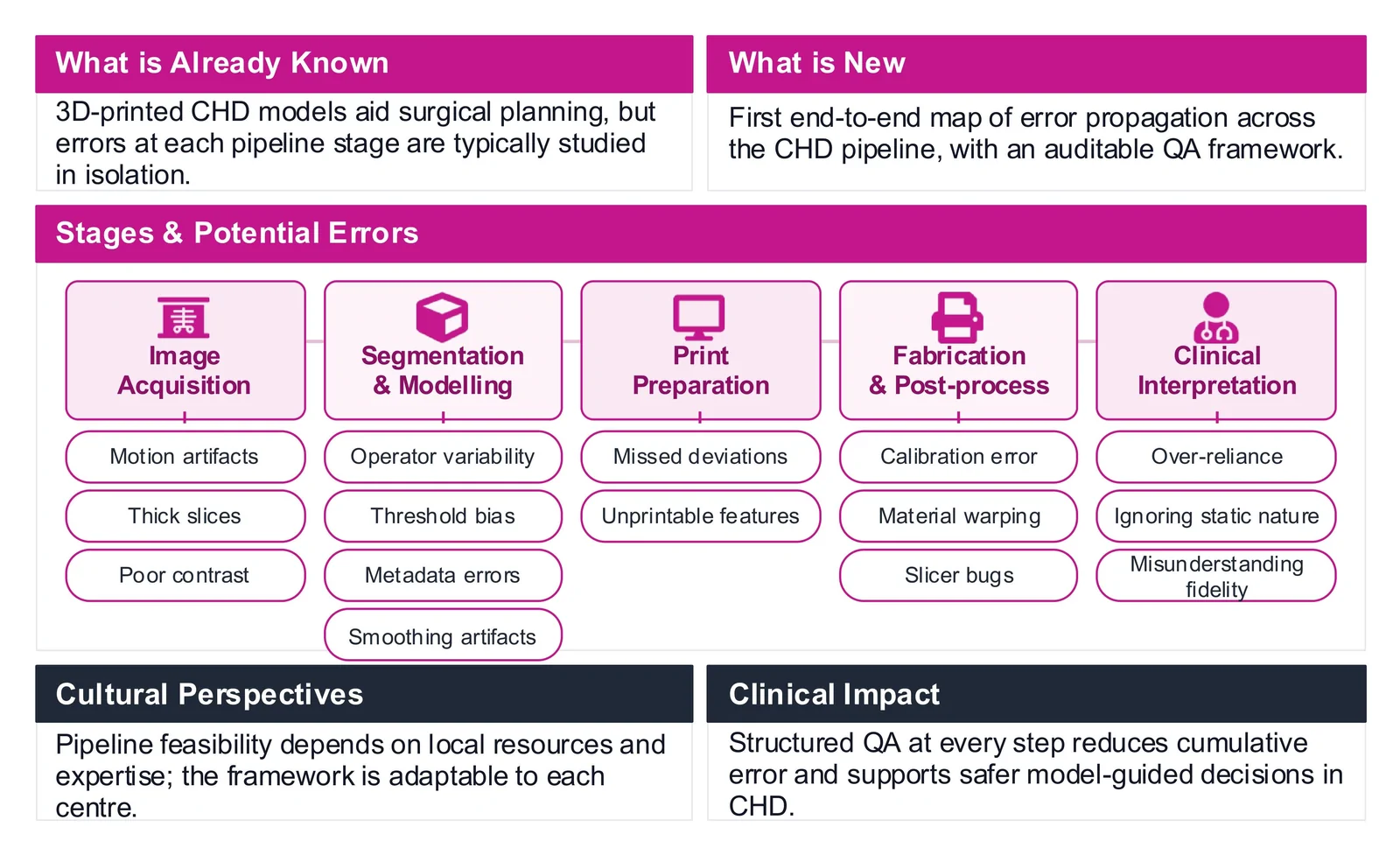

## Background

The application of 3D printing to congenital heart disease (CHD) has advanced from experimental demonstration to practical adjunct in many centres.^1,2^ Printed models can clarify complex morphologies, communicate operative strategies within multidisciplinary teams, and support simulation or rehearsal of selected technical manoeuvres.^3–5^ Despite these benefits, printed models are derived products whose fidelity depends on a chain of transformations. Unlike primary measurements, each transformation—whether converting anatomy into imaging voxels, segmenting masks from greyscale data, generating printable surface meshes, or fabricating a physical model—introduces assumptions and potential distortions. A systematic appreciation of how such distortions arise and propagate is essential if 3D-printed models are to be interpreted with appropriate confidence, particularly in paediatric cardiac surgery, where a discrepancy of even 1 mm may be clinically meaningful.^6^ The modelling process can be split into major segments, from image acquisition all the way to clinical interpretation (*Figure 1*).

**Figure 1:**
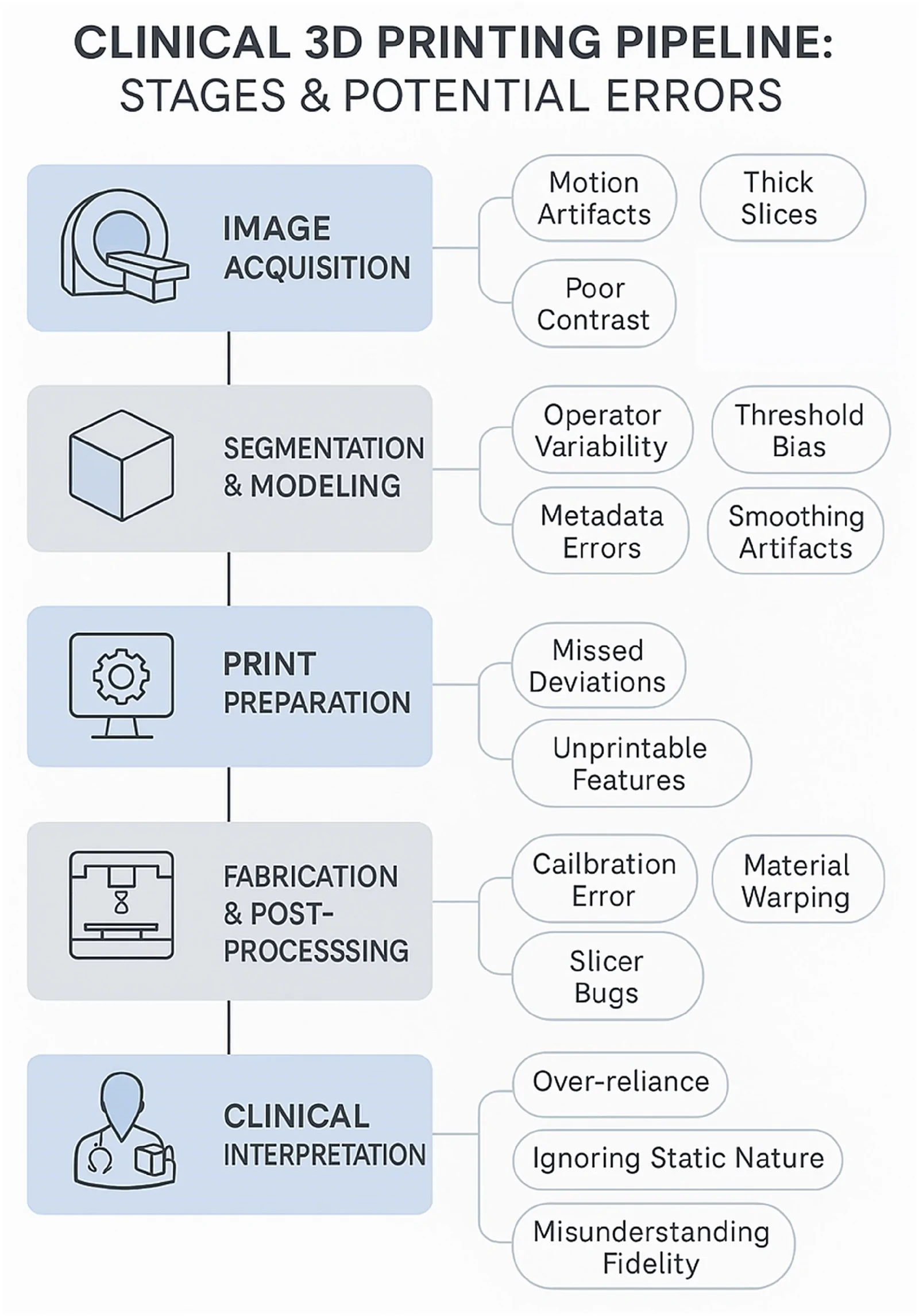
Central illustration: flowchart summarizing different error types for major milestone steps within modelling workflow.

### Image acquisition

Errors commonly originate at the point of image acquisition, where scans are often requested and performed without explicit consideration of segmentation or printing requirements. Slice thickness and reconstruction kernel determine the upper bound of spatial fidelity. Thick slices reduce scanning time and may improve signal-to-noise ratio but, as a consequence, a single imaging pixel (a voxel) often contains multiple tissue types, causing blurring and making edges indistinct. Thin slices enhance detail while inflating file size and magnifying noise, which can complicate segmentation of fine structures such as coronary ostia, valve leaflets, and septal rims.^7^ Paediatric studies present additional challenges related to motion: incomplete sedation, respiratory excursion, and cardiac contraction/relaxation can produce blurring or phase mismatch when electrocardiogram (ECG)-gating is suboptimal.^3,8^ Furthermore, contrast timing and injection techniques demand precision and consideration, as each patient is different, to avoid inadequate opacification of chambers and vascular territories, which would otherwise force operators to infer borders during segmentation. Finally, the frequent practice of archiving only diastolic phases in ECG-gated datasets can obscure dynamic phenomena—e.g. systolic right ventricular outflow tract narrowing, systolic changes in ventricular septal defect effective orifice area, or leaflet coaptation—thereby encoding a phase-dependent bias into the model^4,9^ (*Figure 2*). Imaging modality choice introduces additional trade-offs: computed tomography (CT) generally offers superior spatial resolution at the cost of ionizing radiation exposure and need for intravenous contrast administration, while magnetic resonance imaging provides soft tissue contrast but often requires longer acquisitions, advanced sequences, and meticulous post-processing to support high-fidelity modelling^8^ (*Figure 3*).

**Figure 2:**
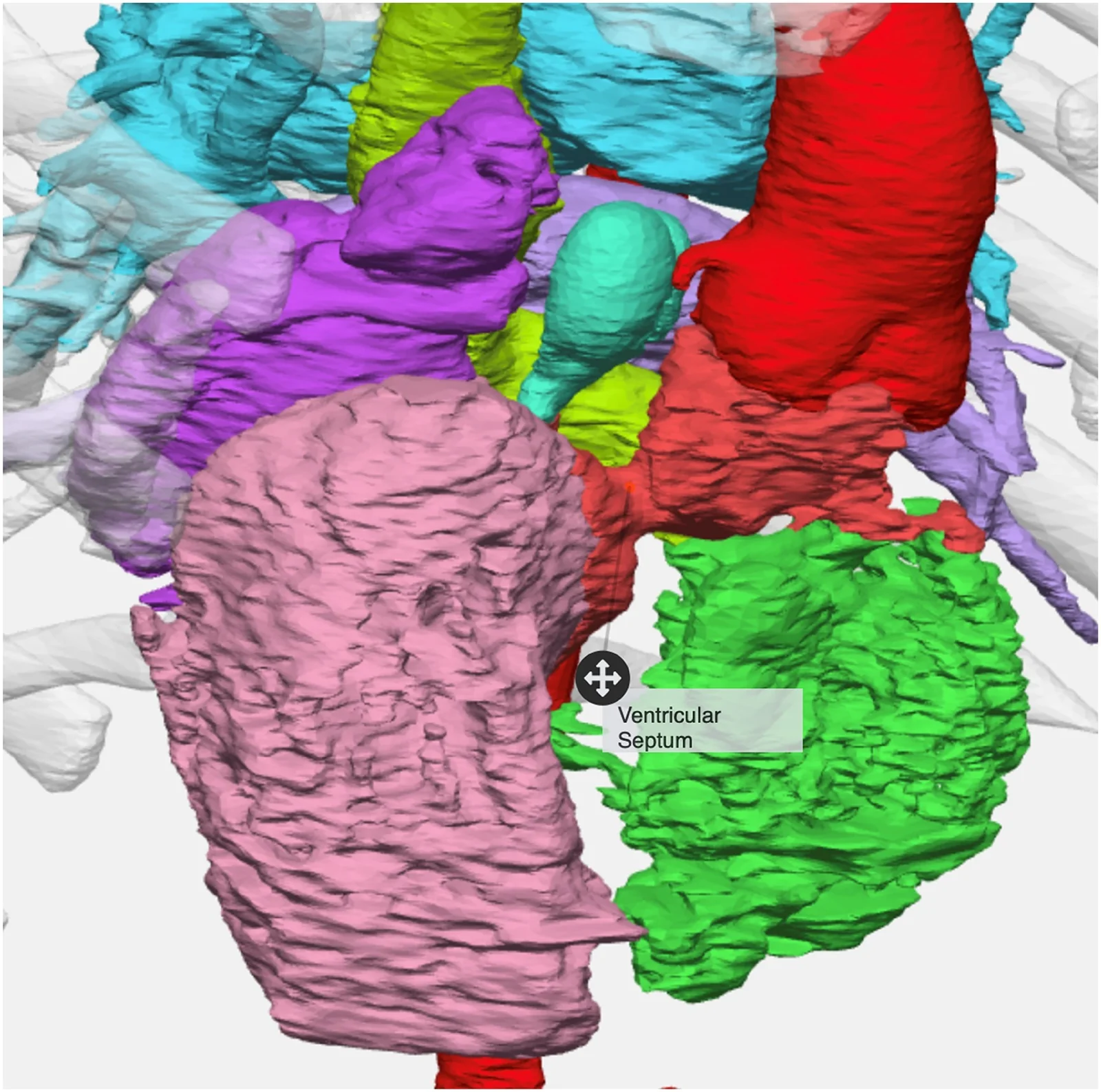
Image showcasing a post-operative Rastelli-Senning patient with dynamic obstruction of the outflow pathway. This model was created from a diastolic image; however, on echocardiography, the pathway was seen to be further narrowed during systole from septal muscle contraction. The empty space with the label highlights where the ventricular septum would be.

**Figure 3:**
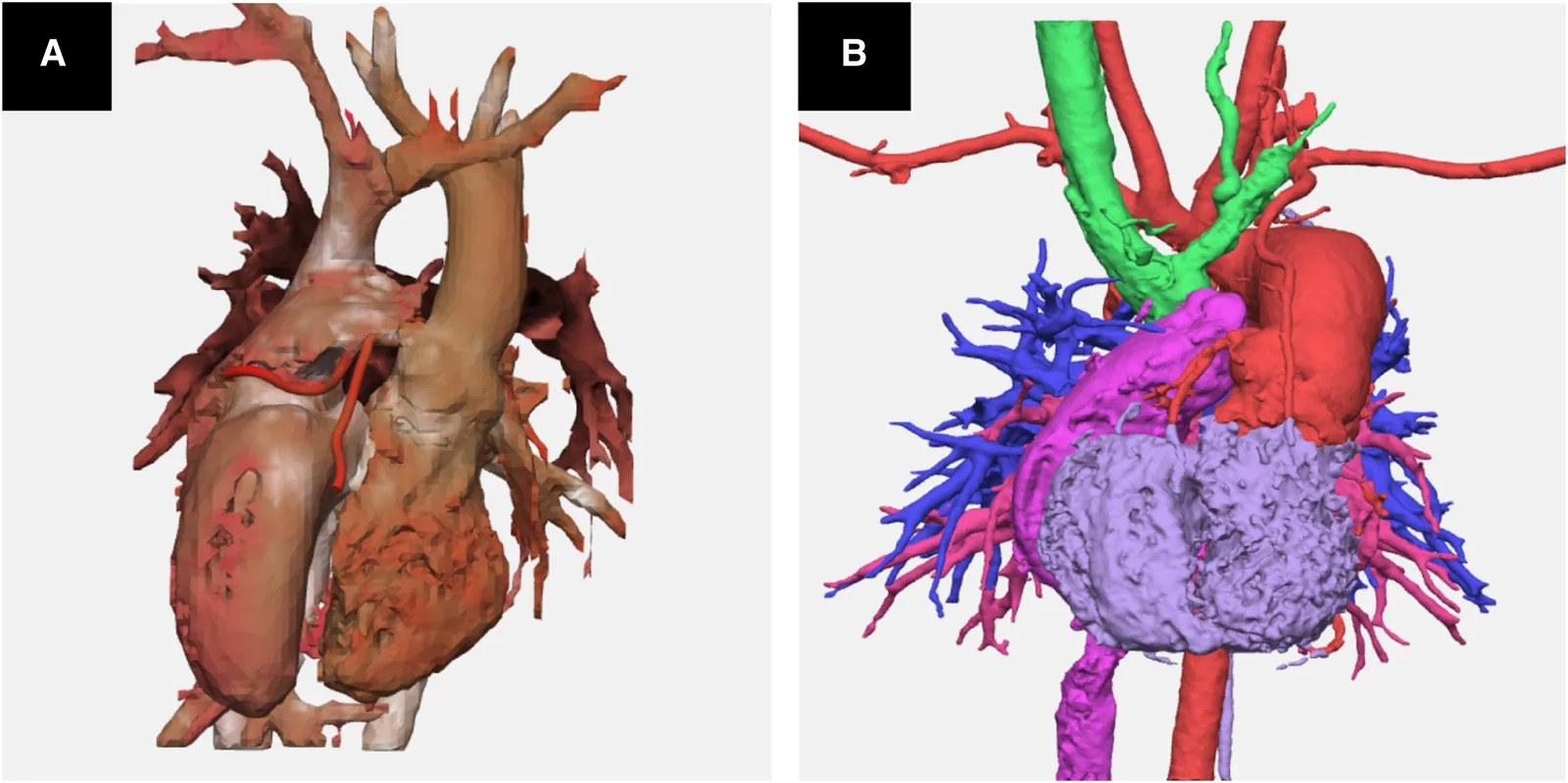
Two segmented images of a patient, one from an MRI (*A*) and one from a CT scan (*B*). It is noticeable that the level of detail in the trabeculations of the ventricles and finer details is more appreciable in the segmentation from the CT scan (*B*).

### Data loss during DICOM creation and export

A frequently overlooked source of error occurs at the level of DICOM creation and export. Even when raw imaging acquisition achieves adequate spatial and temporal resolution, downstream practices in reconstruction, storage, or transfer can irreversibly degrade data fidelity before segmentation begins.

Reconstruction parameters exert a critical influence. CT scanners routinely generate multiple reconstruction kernels from the same raw projection data; ‘soft tissue’ kernels suppress noise but blur edges, while ‘bone’ kernels sharpen boundaries at the expense of introducing noise.^7^ The settings used to create the final images from the raw scan data are critical. For example, a scanner can create a ‘soft’ image that looks smooth but blurs important edges or a ‘sharp’ image that defines edges but looks grainy. If only the ‘soft’ version is saved, important details are lost. Similarly, the choice of slice thickness is decisive: high-resolution acquisitions may be reconstructed at 0.5–0.6 mm, yet the exported DICOMs may be limited to 2–3 mm slice thickness for routine diagnostic review, with permanent loss of fine anatomical detail.^10^ These practices are especially detrimental when modelling small structures such as coronary ostia or small septal fenestrations.

Storage and export protocols may also introduce degradation. Picture Archiving and Communication System utilization of compression or downsampling for long-term storage or for export to portable media can further degrade data quality. Subtle alterations in greyscale gradients can disrupt threshold-based segmentation, particularly at blood–tissue interfaces. Secondary reconstructions (e.g. sagittal or coronal multiplanar reformats) are frequently stored at lower resolution, relying on interpolation rather than original voxel data^11,12^ (*Figure 4*).

**Figure 4:**
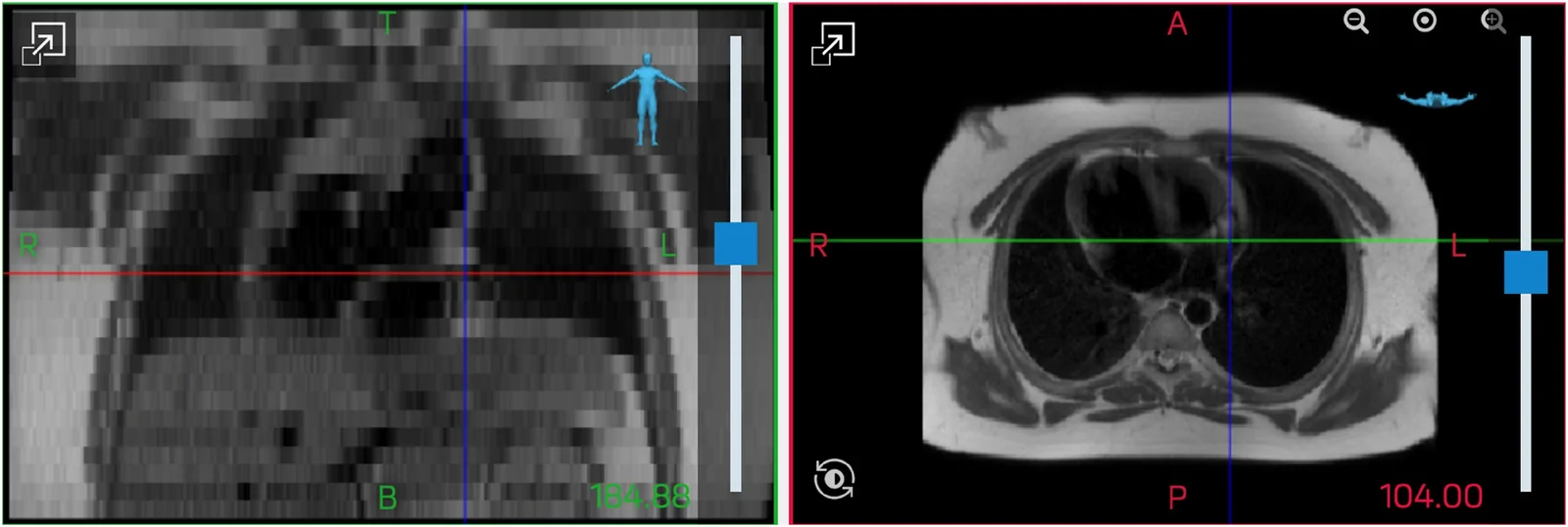
Figure depicting cardiac MRI that showcased a resampling error during DICOM export, leading to poor generation of the coronal view due to the reference image set being only the axial scan.

Errors in metadata and orientation represent an additional pathway for data distortion. Mis-specified pixel spacing or image orientation values within the DICOM header may propagate directly into segmentation software, producing systematic scaling errors of several per cent. Anonymization workflows may further strip or alter metadata required for correct orientation, voxel scaling, and coordinate integrity.^13^

### Segmentation and modelling: operator-dependent variability

Segmentation and modelling represent among the most operator-dependent stages and constitute a major source of inter- and intra-observer variability.^14^ Published CHD-specific segmentation studies help to place a scale on this variability. Inter-observer Dice Similarity Coefficients (DSC) across broader CHD whole-heart segmentation cohorts reported DSC values span ∼0.70–0.95 depending on anatomy, image quality, and operator experience.^14–16^ Even when global DSC is high, small but clinically salient features such as coronary ostia and septal fenestrations may disagree by amounts that could alter operative planning. The final 3D model is heavily influenced by the professional background of the person creating it. An expert imaging cardiologist, for example, is accustomed to interpreting raw scan data and understanding complex spatial relationships, leading them to prioritize overall anatomical accuracy. This differs from a surgeon, who may focus more narrowly on landmarks critical for a procedure, or a biomedical engineer, whose goal is a computationally sound model with a *watertight* surface for downstream analysis like meshing. Less experienced users, meanwhile, are more likely to depend on the software’s initial automated outlines. The required level of precision for a 3D anatomical model is also not absolute but is contingent upon its intended application. This principle, often termed *fitness for purpose*, dictates the necessary investment of time and methodological rigour in the data segmentation stage. For instance, models for educational purposes require only general anatomical accuracy, where overall form is prioritized over fine detail, permitting a low resource allocation. In contrast, models intended for surgical or interventional planning demand the highest degree of geometric and topological accuracy. In this high-stakes clinical context, the meticulous delineation of critical structures and their precise spatial relationships is paramount, as inaccuracies can directly influence procedural success and patient safety. A mismatch between the model’s fidelity and its application can result in either an inefficient allocation of resources or the introduction of clinically significant error. This is especially relevant when the segmentation was requested in a case where there is uncertainty on the ideal treatment pathway and the segmentation is planned to be used to decide upon the planned strategy.

Threshold selection is particularly influential. Overly permissive thresholds risk bridge formation between adjacent tissues and the loss of small clefts; overly conservative thresholds may perforate thin walls or eliminate fenestrations when this may be an area of importance. Manual editing remains a crucial step that introduces subjectivity at points of high leverage, especially where image contrast is limited (*Figure 5*).

**Figure 5:**
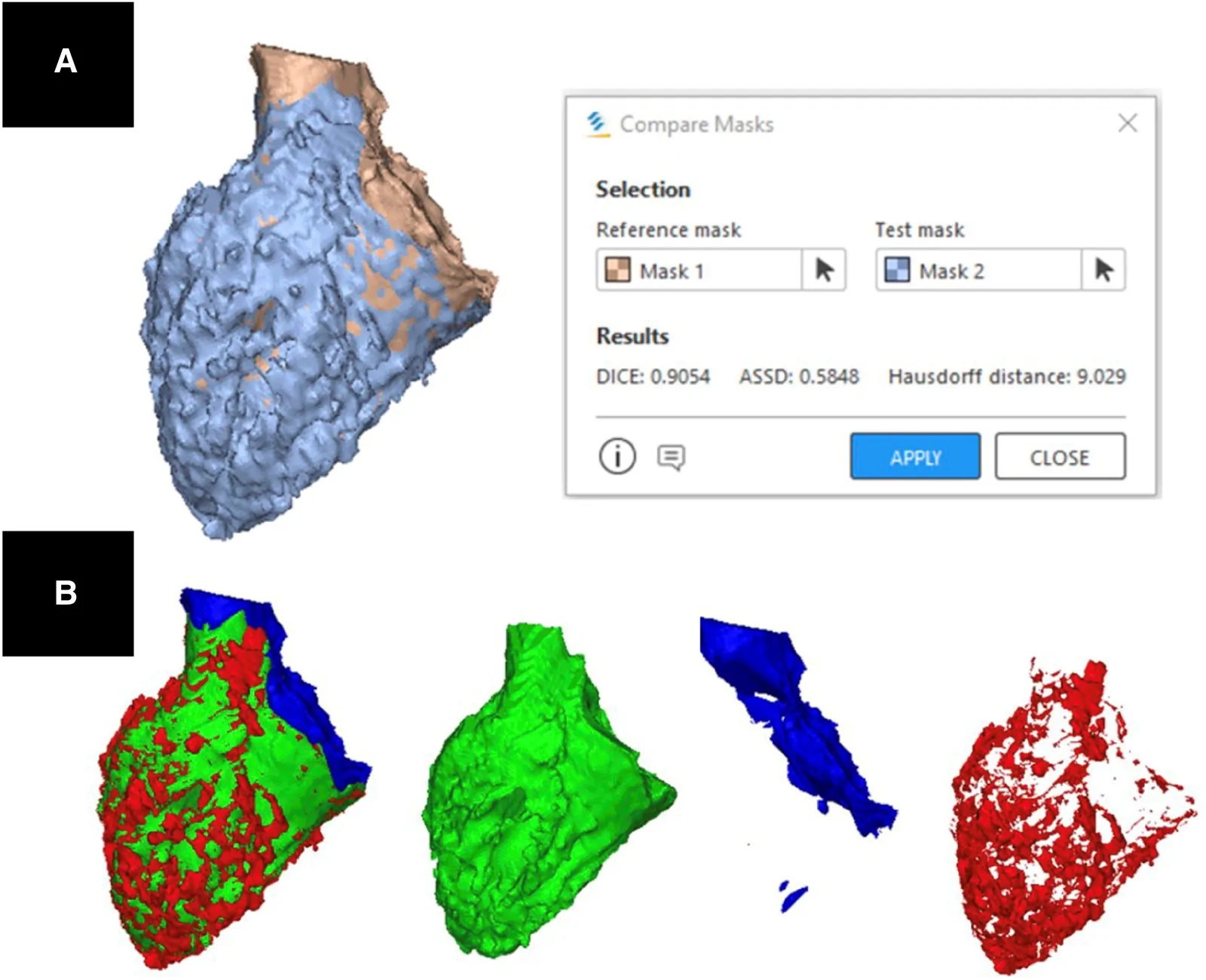
A mask-level comparison of two different operators undertaking a thresholding task for the same image set, with mask 1 as the reference mask from the primary operator and the as the secondary operator (*A*). Panel (*B*) showcases the difference in masks at a visual level, with the green mask indicating the union area of both operators, the blue mask indicating the area under-segmented by operator 2, and the red area indicating the area over-segmented by operator 2.

Software heterogeneity compounds these issues. Commercial and open-source packages differ in DICOM metadata handling, anonymization workflows, axis conventions, and voxel scaling; small inconsistencies at this level can manifest as global scale drift or orientation errors in exported models. While research environments may explore the use of open-source or non-certified software, regulatory-approved medical segmentation software provides standardized algorithms, validated workflows, and audit trails that can mitigate risks. Emerging artificial intelligence (AI)-driven segmentation tools have the potential to further reduce inter-operator variability and shorten processing time, but these still require clinician oversight and robust validation before clinical adoption.^17^ Similarly, AI-based quality assurance systems designed to detect geometric anomalies, mesh errors, or deviations from anatomical norms show promise in safeguarding against unnoticed operator error, though their integration into clinical pipelines remains at an early stage.^18^

Smoothing and decimation improve surface aesthetics and printability but may remove high-frequency geometric content that is clinically relevant, particularly at ostial rims, leaflet edges, or trabecular boundaries. Conversion from volumetric representations to triangulated meshes (e.g. Standard Tessellation Language, or STL) introduces stair-step models, non-manifold edges, and occasional holes; subsequent mesh repair or Boolean operations may infill gaps using geometric rules that do not necessarily reflect anatomy.^19–21^ For example, the software might fill a small hole (like a ventricular septal defect (VSD)) or smooth a rough surface based on a geometric guess, not the patient’s actual anatomy (*Figure 6*). The cumulative effect is a model that appears smooth and coherent but may be incrementally displaced from the underlying anatomical imaging truth.

**Figure 6:**
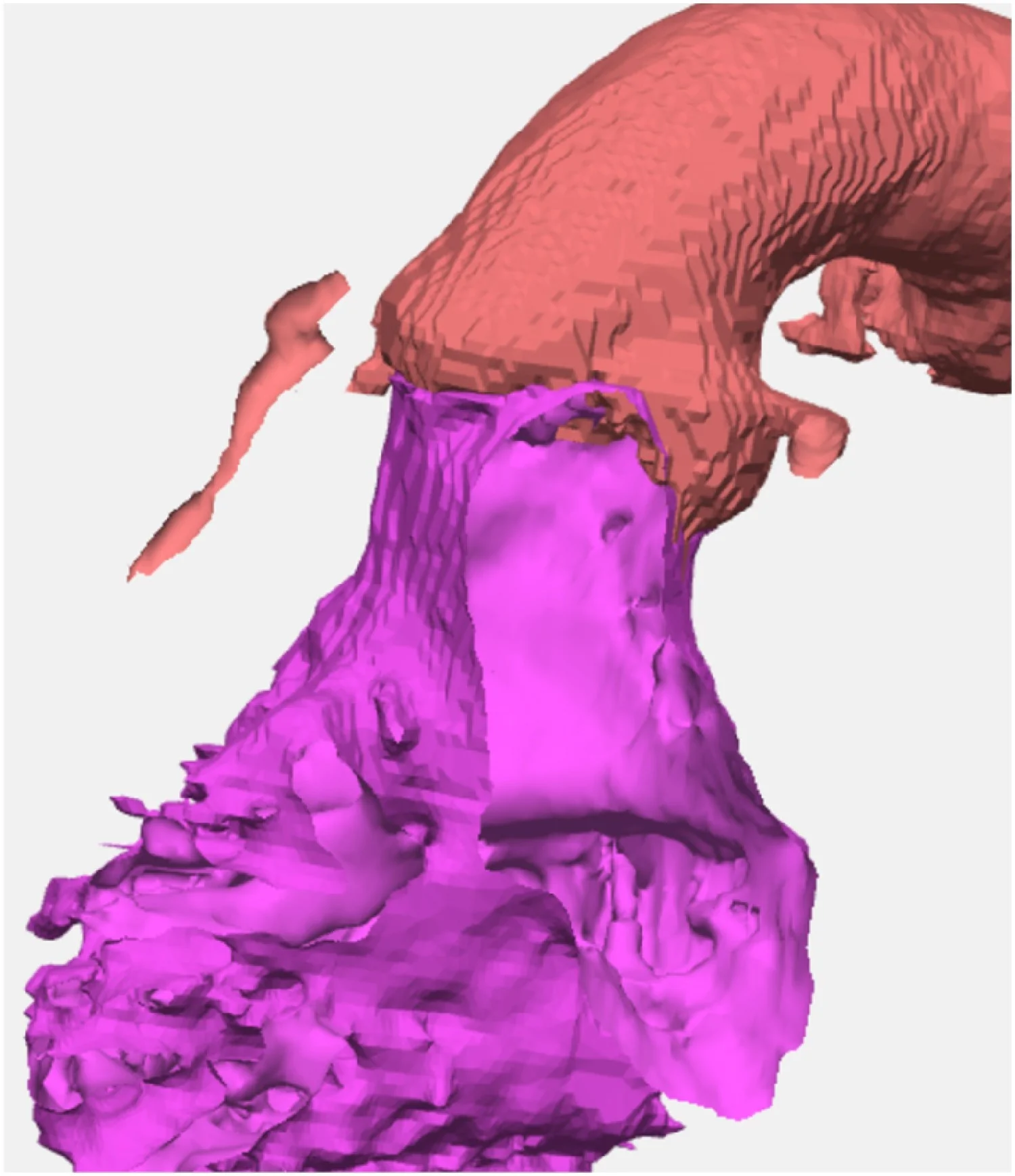
An example of a case with a double outlet right ventricle and ventricular septal defect where, during part generation from the mask, software approximations of the internal surface during hollowing can lead to inadvertent smoothing of the surface, making the defect appear potentially wider than it is.

### Printer-specific processing: hardware and material limitations

During printer-specific processing, the digital surface is translated into a toolpath according to the constraints of the selected technology and material. Slicer software approximates continuous surfaces into layers and trajectories. Layer height constrains vertical resolution, nozzle diameter for fused deposition modelling (FDM) or pixel/voxel size for Vat photopolymerization constrains minimum printable feature size, and default ‘auto-repair’ behaviours may fill small apertures or regularize geometry. Fine anatomical features that approach or fall below these limits are prone to omission or approximation. Calibration errors in the printer can produce systematic scale deviations, while extrusion settings influence wall thickness; over-extrusion enlarges and under-extrusion thins, both of which are consequential for narrowed conduits/tracts and thin septal structures.^22–26^ Retraction and stringing can deform small cavities. Materials contribute additional variability: common FDM polymers (e.g. PLA, ABS) shrink during cooling, with non-uniform warping in thin, curved regions^22,27^; photopolymer resins may exhibit post-cure dimensional changes and material-dependent compliance that may or may not be desirable for the intended simulation.^23–26^ Support structures are often mandatory for complex geometries; however, their removal can damage delicate features or alter local surface quality (*Figure 7*). Post-processing steps such as sanding, infiltration, bonding, or painting can further modify dimensions in ways that are rarely documented quantitatively.^28^

**Figure 7:**
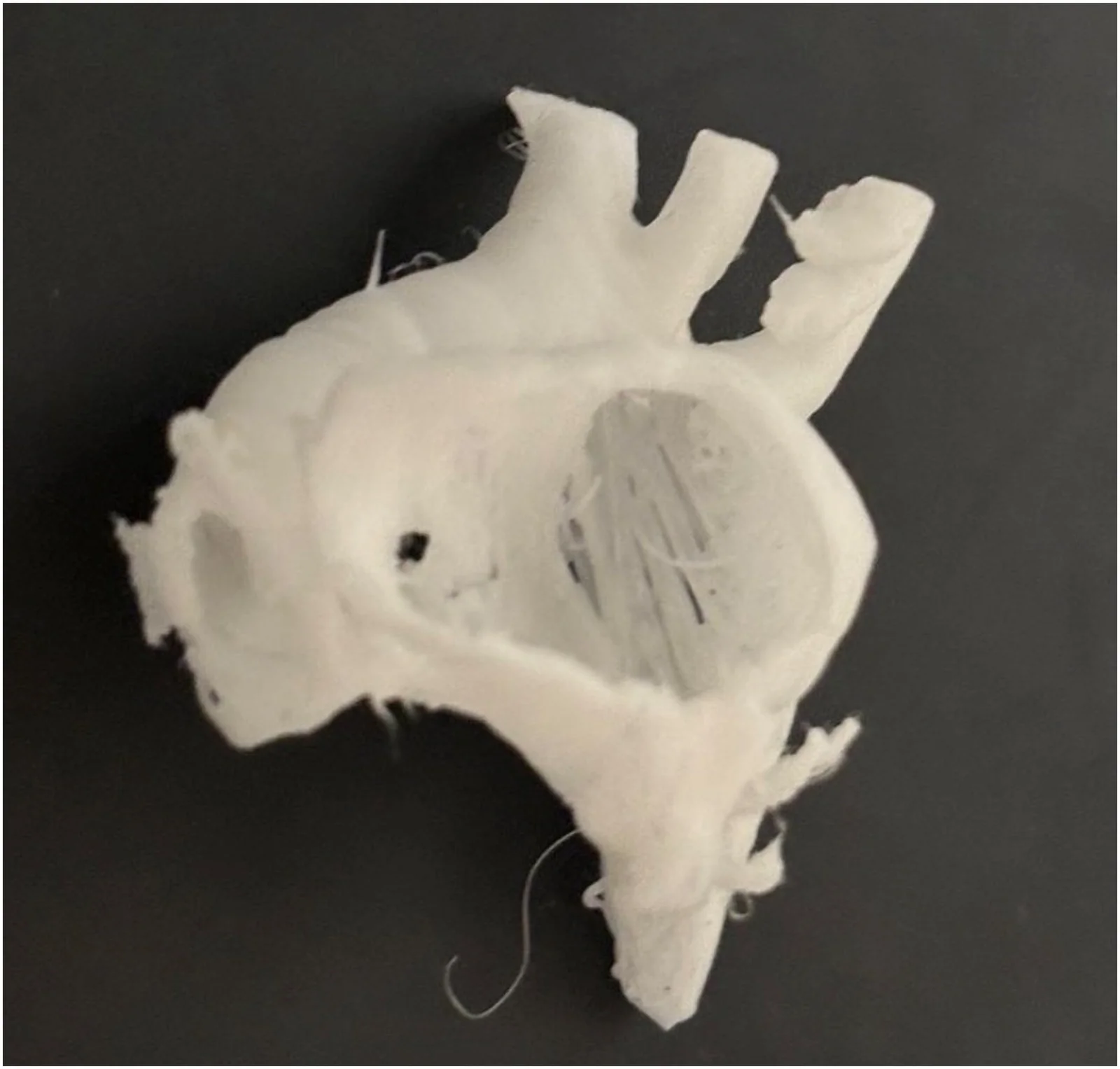
An example of a case where a patent arterial duct and aortic arch were printed from a segmented heart, but during removal of support material from the print, it led to distortion and damage to the walls; stringing and material warping are also seen given the thin nature of the wall in a neonatal segmentation.

### Clinical interpretation

Clinical interpretation is the final stage and, paradoxically, a further source of error when model limitations are not made explicit. Static, single-material models lack pulsatility and do not replicate tissue compliance or pressure-flow interactions; although geometry may be accurate within sub-millimetre ranges in controlled conditions, the absence of physiological behaviour can mislead procedural rehearsal and device sizing.^3,4^ The literature consistently notes high reported accuracy for global dimensions in many series, yet it also documents heterogeneity in workflows, training, and quality assurance across institutions, variables that influence reliability.^5,29^ The clinical significance of this heterogeneity is tangible. In the multi-centre cohort reported by Valverde *et al.*, use of a 3D-printed heart model altered the surgical decision in 19 of 40 complex CHD cases (47.5%), and in three cases reassigned patients from non-surgical to surgical management.^30^ Gomez-Ciriza *et al.* reported a comparable proportion of surgical plans modified after model review,^31^ and Zhao *et al.* reported outcome improvement in patients whose preoperative planning included a 3D model.^32^ Importantly, while these studies demonstrate clinical impact of the model as a whole, the impact of specific stage-level error contributions needs more discussion, so direct quantitative evidence linking a segmentation, STL, or printing error to a changed decision remains limited and is a priority for prospective work. Over-reliance on the visual persuasiveness of a tangible model without due consideration of its derivation can result in misplaced confidence, especially when decisions depend on small margins such as coronary ostial proximity, annular sizing, or the extent of subvalvular obstruction.

### Economic and workflow implications

Fabrication is shaped by the practical trade-offs between print time, costs, and resource availability. High-resolution settings, multicolour printing, and flexible resins allow for finer detail and improved realism but significantly increase production time and material costs. For urgent cases, such long durations are often impractical, prompting the use of faster, lower-resolution settings with a potential loss of accuracy. These pragmatic constraints necessitate a balance between precision and feasibility.

Implementing a robust, error-mitigating pipeline as highlighted in this paper introduces its own costs, primarily in terms of skilled operator time for validation, dual-operator checks, and documentation. However, these upfront investments in quality assurance can be offset by significant downstream value. A high-fidelity model can reduce costly operating room time through enhanced pre-surgical planning, prevent the use of incorrectly sized devices, and potentially avert the need for additional interventions or re-operations. Formal methodologies such as time-driven activity-based costing can be used to quantify this value proposition, framing the additional quality control steps not as an expense but as an investment in clinical efficiency and patient safety.^33^ Any chosen compromise between speed, cost, and fidelity should be transparently documented to contextualize the clinical use of each model.

### Protocol and systems-level error propagation

Taken together, these observations suggest that the sources of error accumulation are largely predictable and can be mitigated. Among the most effective responses are system-level and process-oriented. First, acquisition protocols should be explicitly designed for segmentation and potential printing. This can sometimes be acquired from the segmentation software guidance information. Protocol cards can standardize slice thickness, reconstruction kernels, ECG-gating strategies, and contrast timing appropriate to the suspected pathology. When dynamic behaviour informs decision-making, multiphase datasets, including systolic frames, should be acquired where feasible.^3,8,9^ Secondly, segmentation should follow documented operating procedures that specify Hounsfield unit (HU) ranges by tissue class, permissible manual edits, smoothing parameters, and decimation limits. Version-controlled storage of immutable ‘source masks’ enables independent verification. Where clinical stakes are high, dual-operator segmentation with adjudication by a clinician–engineer panel can reduce subjective drift.^19,20^ Interoperability checks—verifying orientation, voxel-to-millimetre scaling, and metadata integrity across software—are essential when models traverse packages or institutions. Clinically applicable segmentations should also be reviewed by an expert cardiologist or cardiac radiologist.

Before fabrication, a quantitative validation step should compare prioritized linear and areal measurements on the surface model to those obtained from the imaging dataset. Several studies have reported impressive sub-millimetre agreement between printed models and source data when such checks are implemented but have also demonstrated measurable deviations introduced during STL generation and slicing.^24,25^ During printing, routine calibration using dimensional coupons can stabilize scale; slicer ‘auto-repair’ functions should be used judiciously and documented. Material selection should be matched to the intended use (e.g. flexible resins for leaflet manipulation vs. rigid polymers for spatial orientation). After post-processing, targeted calliper measurements of predefined landmarks—or CT of the print with registration to the STL in high-consequence cases—provide an additional safeguard.^24,34^ Finally, transparent documentation should accompany each model. A concise method summary noting acquisition parameters, segmentation thresholds, manual edits, software versions, smoothing/decimation settings, slicer parameters, printer/material, calibration timeline, and validation results allows clinicians to weight confidence appropriately and facilitates reproducibility across centres.

## Discussion

### Quantifying variability: a proposed inter-observer experiment

To quantify the extent and clinical relevance of operator-dependent variability, we propose a compact inter-observer experiment that can be conducted with existing resources as a benchmark periodically to audit the workflow within a centre. An anonymized CT dataset representative of a common planning scenario (e.g. Tetralogy of Fallot with potential coronary anomaly risk) is selected. This should contain at least two annotators, representing distinct backgrounds—for example, a congenital cardiothoracic surgeon or cardiologist (clinical expert), a segmenter with routine segmentation experience (technical expert), and/or a radiologist—who segment independently using the same software environment. Minimal standardized instructions are provided (target structures to include general segmentation aims), but thresholds, smoothing parameters, and manual editing choices are left to operator judgment to reflect typical practice. Each operator exports the mask and STL with a log of key settings.

Agreement is quantified using volumetric DSC (global and structure-specific), Hausdorff distance, and mean surface distance.^20^ A small set of predefined linear measures relevant to clinical decision-making (e.g. minimal right ventricular outflow tract calibre, coronary ostial diameters, longest/shortest ventricular septal defect diameters) are compared. Differences are visualized using colour-mapped surface distance plots. A clinician panel then reviews the models with metrics masked and adjudicates whether measured differences would plausibly alter management (e.g. conduit size selection, feasibility of valve-sparing strategy, cannulation plan). Prior reports indicate that overall, on the model, the Dice similarity may be high, but small clinically salient structures may vary meaningfully across operators, particularly where thin tissue planes and steep gradients challenge thresholding.^19,20^ This design links quantitative divergence to potential clinical consequence and can inform local guardrails—for example, mandatory dual review for structures such as the valve annulus or vessel ostia, threshold ranges for blood-pool extraction, and prohibition of smoothing within a specified margin of critical structures.

### Proposed error-minimizing pipeline

The following pipeline consolidates the preceding recommendations into a practical framework that is implementable in most CHD programmes:

#### Acquisition with intent

Use protocols optimized for segmentation and printing: thin slices at or below the size of critical features, appropriate reconstruction kernels, lesion-specific gating and contrast timing, and inclusion of systolic data when dynamic assessment is clinically relevant.^3,8,9^

#### Disciplined segmentation

Pre-specify HU ranges by tissue; document all manual edits; limit and log smoothing/decimation parameters; preserve immutable source masks; and apply dual-operator verification for high-stakes models.^19,20^ Use of regulatory-approved segmentation software to ensure validated workflows and audit trails. AI-assisted segmentation may reduce inter-operator variability and processing time, but outputs should always undergo thorough clinician review and validation before adoption into clinical decision-making.^35^

#### Pre-print validation

Compare key measurements on the surface model with the imaging ground truth; explicitly identify structures that fall below printer feature size and document any intentional geometric adjustments.^24,25^

#### Calibrated fabrication

Maintain recent printer calibration; use slicer auto-repairs sparingly and record parameters; choose materials according to use-case (rigid orientation models vs. flexible simulation components); and match layer height/nozzle size or resin resolution to the smallest clinically relevant features.^22,23^ Consider trade-offs between print time, cost, and resource availability. Clear documentation of any compromises that may impact clinical interpretation. Evidence indicates that sub-millimetre fidelity is feasible under these conditions.^23–25^

#### Post-print verification

Perform targeted dimensional checks of predefined landmarks; in high-consequence scenarios, a CT scan of the 3D-printed model to be overlaid back on the original image can be used to quantify any deviation.^24,34^

#### Transparent reporting and interpretation

Attach a concise method and quality assurance summary to each model (including segmentation process, use of certified software or AI tools, slicing settings, and material choices); and discuss limitations explicitly during clinical review, including the absence of compliance/pulsatility and any known compromises made for printability.^4,5,36^

This approach emphasizes reproducibility, quantitative verification, and informed interpretation. It does not preclude technological innovation—such as multi-material, compliant models or integrated virtual reality workflows—but seeks to ensure that current practice achieves its best attainable accuracy utilizing available tools.^3,21^

### Realizing shortcomings for specific anatomies

In the evaluation of the 3D modelling pathway, it is crucial to understand ingrained shortcomings of the process simply due to the inability of imaging to capture data at anatomical precision levels. At present, although echocardiography-based segmentation does exist, it is not routine practice in many institutions, and it is still not common across different software. This automatically limits the ability of 3D models to be generated with accurate depictions of the cardiac valves, segments of the subvalvular apparatus, and fenestrations (*Figure 8*). A common misconception made in the interpretation of generated models is that they will provide details superior to conventional imaging; however, this needs to be dispelled. Models provide the details from conventional imaging but re-present them in a different format for ease of interpretation. It does not present additional information. Furthermore, a key limitation that needs to be considered is dynamic changes to the anatomy within the cardiac cycle, such as changes in the size of the aortic root between systole and diastole. When planning patches that necessitate evaluating outflow tracts, it is important to realize that the size of the structure will increase during systole, where the maximum volume of blood will traverse the structure. Similarly, when evaluating VSD dimensions, it is important to note the dynamic behaviour of the ventricular septum that may change VSD size between systole and diastole.

**Figure 8:**
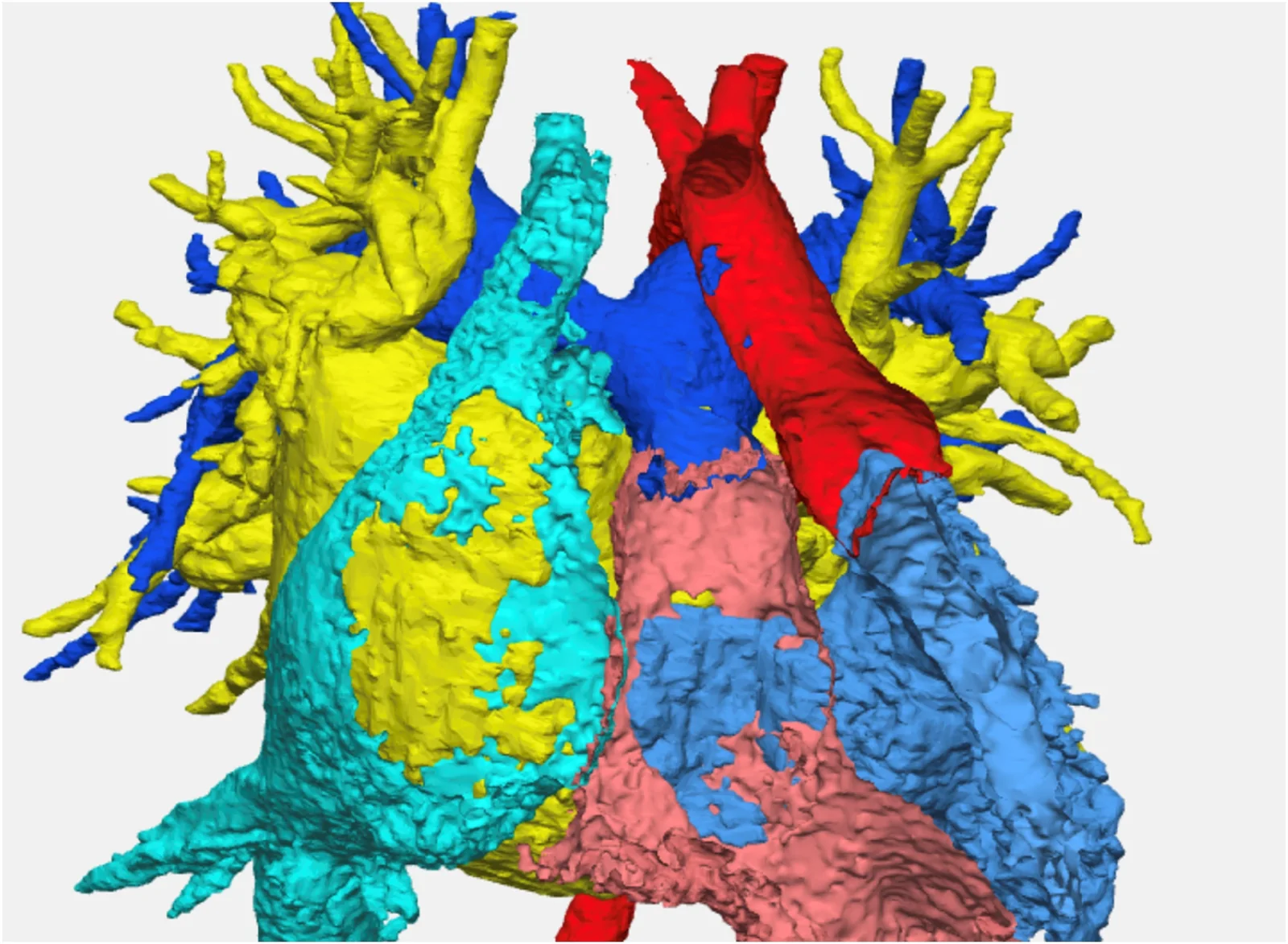
A case of congenitally corrected transposition of the great arteries and ventricular septal defect where there was a question regarding the ventricular septal defect size, relation to the aorta, and a chordal insertion from the left-sided atrioventricular valve. The first two questions could be answered with the segmentation; however, the question of the chordal insertion could not be answered due to the inability to clearly depict the subvalvular apparatus using cross-sectional imaging and the inability to integrate echocardiographic data within the segmentation at the time.

### Limitations

The authors acknowledge several limitations to this analysis. This paper is presented as an integrative review of literature and not a formal systematic review or meta-analysis; as such, it provides a qualitative framework for understanding sources of error rather than a quantitative synthesis of their magnitudes. Comprehensive, stage-by-stage quantification of cumulative error across the end-to-end 3D-printing pipeline for CHD is not currently feasible for three reasons. First, the published literature is heterogeneous, and publication of specific quantitative metrics is limited.^37^ Secondly, error magnitudes depend on local choices of scanner, reconstruction kernel, slice thickness, segmentation software, operator experience, STL export settings, slicer, printer, material, and post-processing; most studies hold some variables fixed and vary others, so their point estimates do not transfer to another centre. Thirdly, the assumptions needed for analysis, including independence of errors at each stage, are not satisfied in practice. For example, segmentation error depends on DICOM export parameters, and slicer auto-repair can mask or amplify STL error. We have therefore chosen to summarize where evidence exists and to flag where it does not, rather than to force a composite numerical estimate that the underlying literature cannot support. The proposed error-minimizing pipeline consolidates best practices but does not account for the wide variability in local resources, software availability, and institutional expertise that will influence its practical implementation. We therefore present it as a conceptual framework for local adaptation. Future multi-centre studies are needed to quantitatively assess the impact of these variables and to validate standardized quality assurance protocols, such as the inter-observer experiment proposed herein.

### Future directions

We intend to further evaluate other potential sources of error and, through implementation of the inter-observer experiment within our centre, seek to build a quantifiable risk of error assessment metric that can be showcased alongside specific cases as an adjunct to the segmentation itself. Alongside this, the authors are in the process of describing a low-resource pathway for segmentation and model generation while considering error propagation within the pathway. Evaluation of pathways against an index is needed to improve standardization of practice in 3D modelling for CHD. Alongside this, we propose the need for software transparency in error propagation at different stages, as factors such as file compression, extrapolation attempts by software, and interpolation of voxels within the DICOM file itself may oftentimes, not be clear to clinicians. The authors aim to conduct a comparative evaluation of segmentation software following a standardized protocol to evaluate the significance of any observed differences.

## Conclusion

3D printing for CHD provides a valuable bridge between complex imaging and operative planning, but its clinical credibility depends on disciplined control of error introduction and propagation across the entire pipeline. Imaging protocols designed for segmentation, structured and auditable segmentation practices, quantitative validation before and after fabrication, calibrated printing with appropriate materials, and transparent reporting collectively reduce variability and enhance interpretability. The recommendations presented here aim to consolidate best practices into a coherent, implementable framework to support reliable clinical use and to lay the groundwork for standardized, multi-centre reproducibility.

## Data Availability

No new data were generated or analysed in support of this research. All sources cited are available via their original publications.

## Lead author biography

Ayush Balaji is a final-year medical student at Hull York Medical School and holds a Master's degree in Clinical Anatomy from the University of Birmingham. His work focuses on 3D segmentation of congenital cardiac anatomy for surgical planning and visualization. He founded the society and serves as Simulation and Surgical Skills Secretary for the Aspiring Congenital Heart Surgeons Association (ACHSA) and sits on the national research committee for cardiothoracics.
